# Effectiveness of a structured stimulated spontaneous safety monitoring of medicines reporting program in strengthening pharmacovigilance system in Tanzania

**DOI:** 10.1038/s41598-022-19884-0

**Published:** 2022-09-27

**Authors:** Kissa W. Mwamwitwa, Adam M. Fimbo, Elias M. Bukundi, Alex F. Nkayamba, Deus Buma, Eva P. Muro, Betty A. Maganda, Danstan H. Shewiyo, Morven C. Shearer, Andrew D. Smith, Eliangiringa A. Kaale

**Affiliations:** 1 Pharm R&D Lab and Department of Medicinal Chemistry, School of Pharmacy, Muhimbili University of Health and Allied Sciences, 65545 Dar es Salaam, Tanzania; 2 Tanzania Medicines and Medical Devices Authority, 77150 Dar es Salaam, Tanzania; 3 Muhimbili University of Health and Allied Sciences, 65001 Dar es Salaam, Tanzania; 4 Department of Pharmacy, Muhimbili National Hospital, 65000 Dar es Salaam, Tanzania; 5 Kilimanjaro Christian Medical University College, 2240 Moshi, Kilimanjaro Tanzania; 6 Department of Pharmaceutics and Pharmacy Practice, Muhimbili University of Health and Allied Sciences, 65013 Dar es Salaam, Tanzania; 7 School of Medicine, University of St. Andrews, Fife, KY16 9TF Scotland, UK; 8 School of Chemistry, University of St. Andrews, Fife, KY16 9TF Scotland, UK

**Keywords:** Health care, Medical research

## Abstract

Under-reporting of adverse drug events (ADEs) is a challenge facing developing countries including Tanzania. Given the high magnitude of under-reporting, it was necessary to develop and assess the effectiveness of a ‘structured stimulated spontaneous safety monitoring’ (SSSSM) reporting program of ADEs which aimed at strengthening pharmacovigilance system in Tanzania. A quasi-experimental design and data mining technique were used to assess the effect of intervention after the introduction of program in seven tertiary hospitals. ADEs reports were collected from a single group and compared for 18 months before (July 2017 to December, 2018) and after the program (January 2019 to June 2020). Out of 16,557 ADEs reports, 98.6% (16,332) were reported after intervention and 0.1% (23) death related to adverse drug reactions (ADRs) were reported. Reports increased from 20 to 11,637 after intervention in Dar es salaam, 49 to 316 in Kilimanjaro and 17 to 77 in Mbeya. The population-based reporting ratio per 1,000,000 inhabitants increased from 2 reports per million inhabitants in 2018 to 85 reports in 2019. The SSSSM program can increase the reporting rate of ADEs and was useful in detecting signals from all types of medicines. This was first effective developed spontaneous program to monitor medicine safety in Tanzania.

## Introduction

Adverse drug events (ADEs) presents a major global health problem that contributes to the increased morbidity, mortality, and health care cost^1,2^. ADEs refers to any negative or harmful occurrence that takes place during treatment that may or may not be associated with a medicine^1^. It encompasses both harm that results from intrinsic nature of a medication (an adverse drug reaction, ADRs) as well as harm that results from medication errors^3^. ADRs have been defined by the World Health Organization (WHO) as “a response to a drug which is noxious and unintended, and which occurs at doses normally used in man for the prophylaxis, diagnosis, or therapy of disease, or for modification of physiological function”^4,5^. ADRs are one of the mounting causes of morbidity and mortality, and reported to continue to be a significant public health issue^5^. It is estimated that ADRs caused deaths in 0.02% to 5% of all hospital admissions and about 1.7% to 11.9% of the admissions are due to ADRs^6–8^. Detection of rare and sometimes severe adverse events require evaluation of a large number of exposures which can be obtained in the pharmacovigilance databases^9,10^. Spontaneous, sometimes known as passive or voluntary reporting of suspected ADEs, represents the backbone of pharmacovigilance and post-marketing signal detection^6,11,12^. Pharmacovigilance can be used to identify signals that may be novel in their nature, severity, and or frequency^13^. However, under-reporting of ADEs, quality of reported data, and lack of information on drug exposure are amongst the challenges facing pharmacovigilance systems in African countries including Tanzania^11,14^. It is estimated that overall ADEs reporting represents not more than 5–10% of all ADEs^11,15^. Under-reporting reduces the sensitivity of the reporting systems and delays signal detection^6,16,17^.

In pharmacovigilance practice, among other reporting methods, the spontaneous reporting is mostly used ^11,12^. It allows rapid detection of potential signals through the early detection of new adverse drug reactions (ADRs)^14^. The involvement of various healthcare professionals (HCPs) such as physicians, nurses, clinical pharmacists in spontaneous reporting helps in identification of events with low frequency^11^. Despite, the involvement of HCPs in ADRs reporting, under-reporting still exist^11,14^.

Several studies have shown a positive effect of educational interventions on the improvement of reporting of ADRs through a change of HCPs behaviours/attitude towards ADRs reporting^6,17,18^. However, most of the studies assessing the impact of an educational intervention on improvement of ADRs reporting were conducted in high-income countries. Due to the differences in infrastructures, capacities, disease distribution, culture, medical education program, and economic status, the finding from these studies cannot be generalized in developing countries such as Tanzania.

In Tanzania, pharmacovigilance systems^19^ had been implemented since 1989 through the spontaneous reporting system^20,21^. The system uses specially designed forms (“Yellow Forms”) and electronic platforms such as mobile phones and computers to collect ADEs from patients^21^. The systems are coordinated by the Tanzania Medicines and Medical Devices Authority (TMDA) together with established zone pharmacovigilance centers.

Different initiatives have been implemented to strengthen pharmacovigilance systems in Tanzania. These include the use of registries and integration of active monitoring in public health programs. TMDA has conducted capacity building of HCPs to increase awareness of medicines safety monitoring, established pharmacovigilance regulations^19^ and engagement of medical universities and different stakeholders from different institutions^22^. Other approaches were the establishment of more pharmacovigilance centers, sensitizing Regional and Council Health Management Teams and other stakeholders to uphold their responsibilities in pharmacovigilance activities. Similarly, more emphasis has been put in providing prompt feedback from TMDA to reporters.

Despite all these initiatives which have been implemented since the inception of the pharmacovigilance system in 1989, under-reporting is still a challenge that needs to be addressed. In response to this, TMDA has been trying to devise measures to bolster the reporting rate. The establishment of the “Structured Stimulated Spontaneous Safety Monitoring” (SSSSM) method (See next section), an improved spontaneous ADEs reporting method was aimed at assisting reporting of ADEs during safety monitoring of the most commonly used chiral medicines (medicines whose active pharmaceutical ingredient has an asymmetric carbon) in Tanzania^23^. The method was also intended to strengthen the pharmacovigilance system. Based on these considerations, the current study aimed at developing and assessing the effectiveness of the SSSSM program on reporting of ADEs in Tanzania. The evidence-based information generated by this study can be used by medicines regulatory authorities in safety monitoring of medicines and policy formulation to ensure safer practices and improve the quality of patient care. Similarly, findings from this study will provide impetus for regional pharmacovigilance centres and healthcare facilities to adopt the SSSSM intervention to reduce under-reporting of ADEs.

### Development of SSSSM reporting program

The SSSSM program, was a uniquely initiated program designed for safety monitoring of the most commonly used chiral medicines. In order to strengthen pharmacovigilance system as a whole, the program also included all other medicines in spontaneous reporting. The program was developed at the end of December, 2018 by conducting a short training on PV to pharmaceutical staff and other healthcare providers such as nurses and clinicians. The training package was developed first and included contents such as introduction and definition of pharmacovigilance, definition of AEs, ADRs, and how to identify them, types of ADRs, and the yellow forms used to report ADRs. Explanation of the reporting process, and how to enter ADRs data into vigiflow was also taught. Staff were oriented on tools like vigiflow and systematic rotational detection and collection of ADRs among in-patients. The teaching–learning aids used during training were PowerPoint presentations, lectures, posters. The training curricula were undertaken by researchers trained and working in pharmacovigilance centres. The program was conducted in collaboration with seven tertiary hospitals.

The health facility leaders or administrators were explained the purpose of the program and agreed on its implementation before the start of the training. The primary aim of the training was to enhance awareness and improve knowledge among healthcare providers on pharmacovigilance, and ADEs reporting process and hence boost the reporting attitude.

During the implementation of the program, systematic rotational detection and collection of ADRs among in-patients were arranged to pharmaceutical staff (structured). Patients were reached out through phone calls (stimulated) from trained staff after completion of the prescribed regimen to identify new events, stimulated self-awareness, and encouraging reporting of ADEs.

## Materials and methods

### Study setting

This study was conducted in seven (7) tertiary hospitals hosting pharmacovigilance centres located at Muhimbili National Hospital (MNH) in Dar Es Salaam, Kilimanjaro Christian Medical Center (KCMC) in Kilimanjaro, Bugando Medical Centre (BMC) in Mwanza, Ligula Regional Referral Hospital (LRRH) in Mtwara, Dodoma Regional Referal Hospital (DRRH) in Dodoma, Kitete Region Referral Hospital (KRRH) in Tabora, and Mbeya Zone Referral Hospital in Mbeya. Selection of the study sites was based on the existence of the established PV centres.

### Study design

#### Effectiveness of SSSSM reporting program

A quasi-experimental design and data mining technique were used to assess the effectiveness of SSSSM program. The study used a single group before-and-after intervention conducted from January 2019 through June 2020. We compared data of individual case safety reports (ICSRs) (also known as ADEs reports or ADRs reports) for 18 months before the program between July 2017 to December 2018 and 18 months after the program (January 2019 to June 2020). We chose to use the before and after design due to its simplicity and robustness compared to observational research. Also, because the introduction of our intervention may improve reporting rate of ADEs, we decided to use this design to ensure that the control group also benefits from the acquisition of knowledge regarding ADEs reporting and drug safety assessments through the intervention.

### Study participants

The study participants included HCPs in the tertiary hospitals (pharmacy personnels, physicians, nurses, auxiliary nursing and administrative officers). The exclusion criteria included professionals who were on sick leave or vacation.

### Statistical analysis

The ICSRs data were generated in excel from VigiLyze/ Vigibase database and grouped into two groups; data received from 1st July 2017 to 31 December 2018 and from January 2019 to June 2020. Demographic and baseline characteristics were summarized using descriptive statistics. Continuous data were presented as mean ± standard deviation (SD) or median (25–75 percentile) as appropriate, while ordinal data were expressed as number (percentage). The chi-square and Fisher's exact tests were used to compare the difference of received ICSRs before and after the SSSSM program. The significance *P*-value for significant test was < 0.05.

#### SSSSM Program assessment

The effectiveness of the program was measured by:

##### Assessing the number of ICSRs before and after the SSSSM program

The number of ICSRs before and after the SSSSM program was assessed. Additionally, we calculated the population based reporting ratio (PBRR) before and after the program. This was calculated as the total number of ADR reports collected in a safety database per year per million inhabitants.

##### Mapping geographic differences before and after the SSSSM Program

 The number of ICSRs was mapped for each region to identify geographic differences before and after the training program. The frequency of ICSRs for each region before and after the SSSSM program were exported from Stata into Microsoft Excel then imported into Quantum Geographic Information System (QGIS) Version 3.10A Coruna for mapping.

##### Assessing the outcome of the program by identifying signals

The outcome of the program was further measured by (a) assessing if the method was able to detect signals from a newly introduced medicine, Dolutegravir (DTG)-based regimen. In 2018, Tanzania recommended a DTG-based regimen containing Tenofovir/Lamivudine/Dolutegravir (TLD) as the preferred default first-line regimen for adults living with HIV^25^. However, there is currently scarce information about the safety of DTG-based combination regimen, including the types and frequency of suspected ADEs, experienced and reported in Tanzania. Likewise, the outcome of SSSSM program was also measured by (b) assessing if the method was able to detect signals from any other medicine. Medication errors were also included.

Reporting odds ratios (RORs) were used to identify a statistical association between a DTG-based combination regimen, and vancomycin injection adverse events (signals)^26,27^. We calculated 95% confidence intervals (CIs) for RORs using Woolf’s method. Specific ADRs with at least five (5) cases of DTG-based regimen or Vancomycin injection-related ADRs were considered as a suspect for signals associated with adverse reactions and were included in the analysis. A signal was considered to be present if it meets two criteria: minimum of five cases of DTG-based regimen, or vancomycin injection-related ADRs, and if the lower bound of the 95% two-sided confidence interval of the ROR exceeds 1^28^. A two-sided significance level of 5% was considered throughout the analysis. Signals evaluation was done following procedures described elsewhere^29^. Briefly, we compared the identified signal with the already known DTG-based regimen, or vancomycin injection drug events using the manufacturer package leaflets to eliminate ADRs already reported onto the leaflet. The residual signals were submitted to four clinicians to identify signals considered to be rare events deserving further analysis. These clinicians eliminated ADEs that were more likely caused by the disease or other concomitant medications.

### Ethical consideration

Ethical clearance was granted by the National Institute for Medical Research, Tanzania (Certificate number NIMR/HQ/R.8a/Vol.IX/3086) and the Institutional Review Board of Muhimbili University of Health and Allied Sciences (MUHAS) (certificate number DA.282/298/01.C). Approval letters were obtained from each participated pharmacovigilance centres before the start of the study. In this study the informed consent for healthcare professional was not applicable due to the fact that, reporting of AEs is part of daily clinical practice and is mandatory as per pharmacovigilance regulations. The research methods were carried out in accordance with relevant guidelines and regulations pertaining to safety monitoring of medicines in Tanzania.

## Results

### Number and characteristics of ICSRs

The social demographic characteristics for the ICSRs are summarized in Table 1. A total of 16,557 ICSRs were reported between July 2017 and June 2020 and more than half of the patients were aged between 20 and 59 years (68.3%). After assessment, it was found that among the received ICSRs, 15,959 (96.4%) reports had non-serious ADRs, while only 598 (3.6%) had serious ADRs. A total of 23 (0.1%) deaths related to ADRs were reported. Most patients, 13,569 (82%) had recovered at the time of reporting and pharmacist contributed 16,332 (92.8%) of all reports. MNH contributed to 11,436 (69.1%) of all ADRs reports. Number of ICSRs reported by Professionals and institutions are shown in Table 2.

**Table 1:** Social demographic characteristics for ICSRs received between July 2017 and June 2020.

Characteristics	Number of ICSRs	Percentage
**Sex**		
Male	7647	46.2
Female	8091	48.9
Sex not indicated	819	4.9
**Age group (years)**		
< 1	257	1.6
1–9	2270	13.7
10–19	679	4.1
20–59	11,312	68.3
≥ 60	1430	8.6
Age not indicated	609	3.7

**Table 2:** Number of ICSRs reported by Professionals and institutions between July 2017 and June 2020.

Characteristics	Number of ICSRs	Percentage
**Reporters professional**		
Pharmacist	15,360	92.8
Physician	244	1.5
Other health professionals	364	2.2
Custommer	46	0.3
Reporter professional not indicated	543	3.2
**Reporting institution**		
MNH	11,436	69.1
KCMC hospital	291	1.8
Muhimbili orthopaedic institute	150	0.9
Mbeya zonal referral hospital	58	0.4
Kibong'oto hospital	52	0.3
Ocean road cancer institute	36	0.2
Mount meru hospital	27	0.2
Tanga region referral hospital	42	0.2
Tosamaganga hospital	20	0.1
Other health facilities	299	1.8
Reporting institution not indicated	4146	25.0

**Figure 1:**
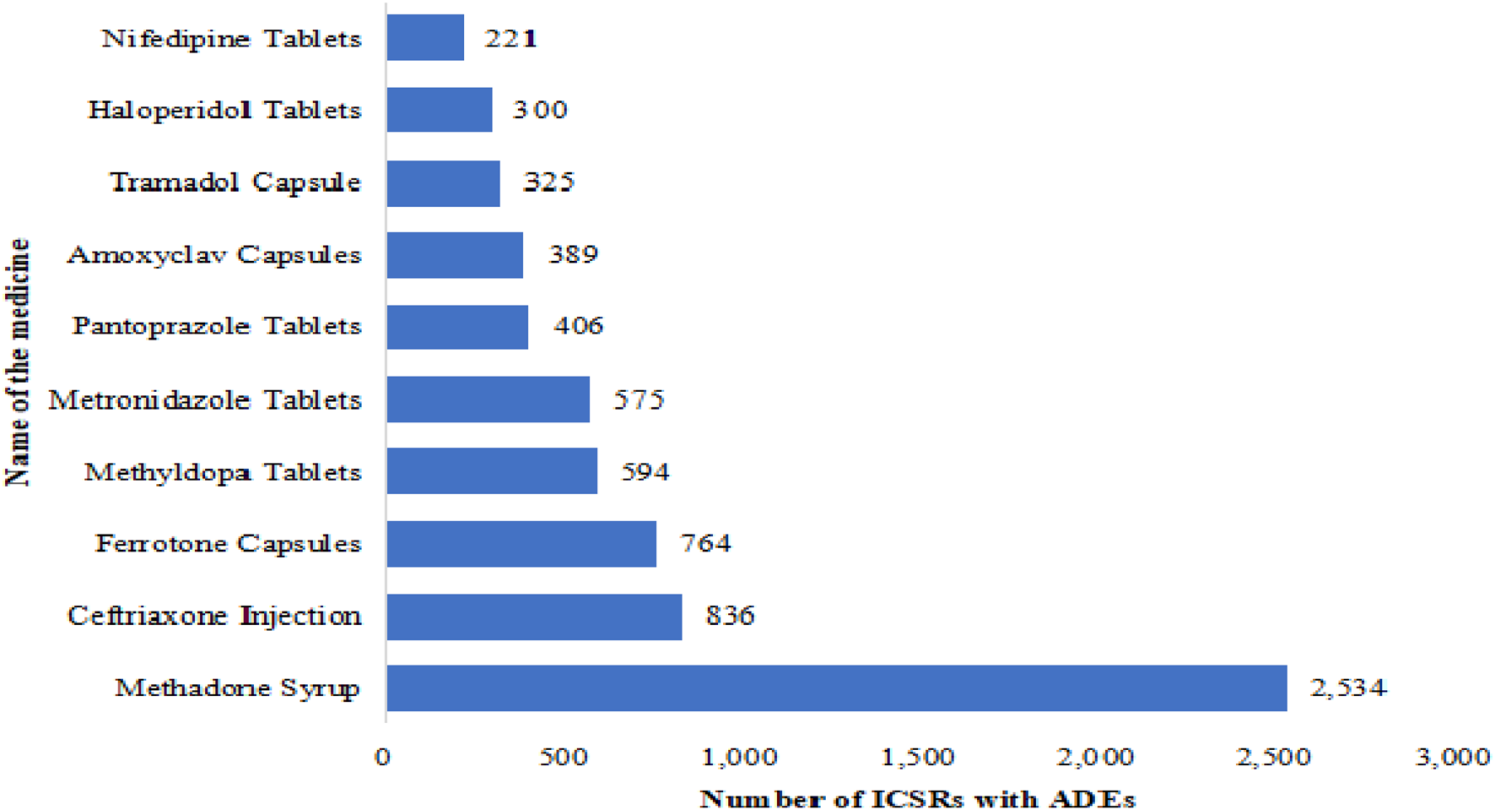
Top ten medicines implicated to cause ADEs between July 2017 and June 2020.

Methadone in syrup form was the most reported medicine (2,534) to cause ADEs followed by ceftriaxone injection (836), ferrotone capsules (764), methyldopa tablets (594), and metronidazole tablets (575). Out of the top ten reported medicines with ADEs, 60% (6/10) were chiral medicines (methadone, ceftriaxone, methyldopa, pantoprazole, amoxiclav and tramadol)^23^. The top ten medicines represented 41.9% (6,944) of all reported ICSRs (Fig. 1).

### Number of ICSRs reported with ADEs before and after the SSSSM program

Generally, in all variables a statistically significant (*P* < 0.05) improvement was seen on reporting comparing the number of reports before and after introduction of the SSSSM program (Table 3). Among the seven (7) regions that were introduced with SSSSM program, the highest reporting improvement was observed in Dar es Salaam, Kilimanjaro, Mbeya, and Mwanza region. There was an increase from 20 ADEs reports before SSSSM to 11,637 reports after SSSSM in Dar es salaam. Also, ADE reports increased from 49 to 316 reports before and after the SSSSM program respectively in Kilimanjaro and 17 to 77 reports in Mbeya. Nevertheless, among the reporting site, the highest improvement was observed at MNH, KCMC, and Muhimbili Orthopaedic Institute. The number of ADEs reports increased from 6 to 11,430 reports, 1 to 290 reports, and 0 to 150 reports at MNH, KCMC, and Muhimbili Orthopaedic Institute respectively. Likewise, a statistically significant improvement (*P* < 0.05) in reporting was observed among pharmacists whereby the number of ADEs reports increased from 87 to 15,273 reports after SSSSM. Surprising, there was no improvement on number of ADEs reports at Dodoma general hospital, Kitete regional hospital and Ligula regional hospital despite being among the regional pharmacovigilance centres trained and sensitized on SSSSM program. Table 3 shows comparison of number of ADEs reports received from different regions and sites before and after SSSSM Program.

**Table 3:** Comparison of number of ADEs reports received from different regions and sites before and after SSSSM Program.

Characteristics	Total	Before SSSSM	After SSSSM	*P* value
		N (%)	N (%)	
**Sensitized region**	Reports	Reports	Reports	
Arusha	30	9 (30.0)	21 (70.0)	0.042
Dar es salaam	11,657	20 (0.2)	11,637 (99.8)	< 0.001
Kilimanjaro	365	49 (13.4)	316 (86.6)	< 0.001
Mwanza	36	3(8.33)	33 (91.67)	< 0.001
Mbeya	93	17 (18.3)	76 (81.7)	< 0.001
Tanga	32	4 (12.5)	28 (87.5)	0.001
Pwani	50	16 (32.0)	34 (68.0)	0.016
Dodoma	10	6 (60)	4 (40)	0.535
Tabora	8	7 (87.5)	1 (12.5)	0.089
*Mtwara	3	0 (0)	3 (100)	–
**Interventional reporting site**				
*Tanga regional referral hospital	23	0 (0)	23 (100)	–
*Bugando medical center	5	0 (0)	5 (100)	–
*KCMC	291	1(0.3)	290 (99.7)	–
*Mbeya zonal referral hospital	58	0(0)	58 (100)	–
Muhimbili national hospital	11,436	6 (0.1)	11,430 (99.9)	< 0.001
*Muhimbili orthopaedic institute	150	0(0)	150 (100)	–
Ocean road cancer institute	36	3(8.3)	33 (91.7)	< 0.001
*Mawenzi regional referral hospital	11	0 (0)	11 (100)	–
Mount Meru hospital	27	9 (33.3)	18 (66.7)	0.099
*Tumbi regional referral hospital	12	1 (8.3)	11 (91.7)	–
*Dodoma regional referral hospital	4	0 (0)	4 (100)	–
*Kitete regional referral hospital	1	0 (0)	1(100)	–
*Ligula regional referral hospital	2	0 (0)	2(100)	–
**Reporter professional**				
Pharmacist	15,360	87 (0.6)	15,273 (99.4)	< 0.001
Consumer/Non-health professional	46	17 (36.9)	29 (63.1)	0.086
Other health professionals	364	71 (19.5)	293 (80.5)	< 0.001
Physician	244	36 (14.7)	208 (85.3)	< 0.001

*Insufficient observations to calculate the *P*-values.

### Population based reporting ratio—PBRR

Out of all 16,557 ICSRs, over 95%, 16,332 (98.6%) were reported after SSSSM implementation. PBRR per 1,000,000 inhabitants increased from 2 reports per million populations of inhabitants in 2018 (120 ADEs reports, 1 year before SSSSM) to 85 reports per million inhabitants in 2019 (5099 ADEs reports, 1 year after SSSSM).

### Mapping of geographical differences before and after the SSSSM Program

**Figure 2:**
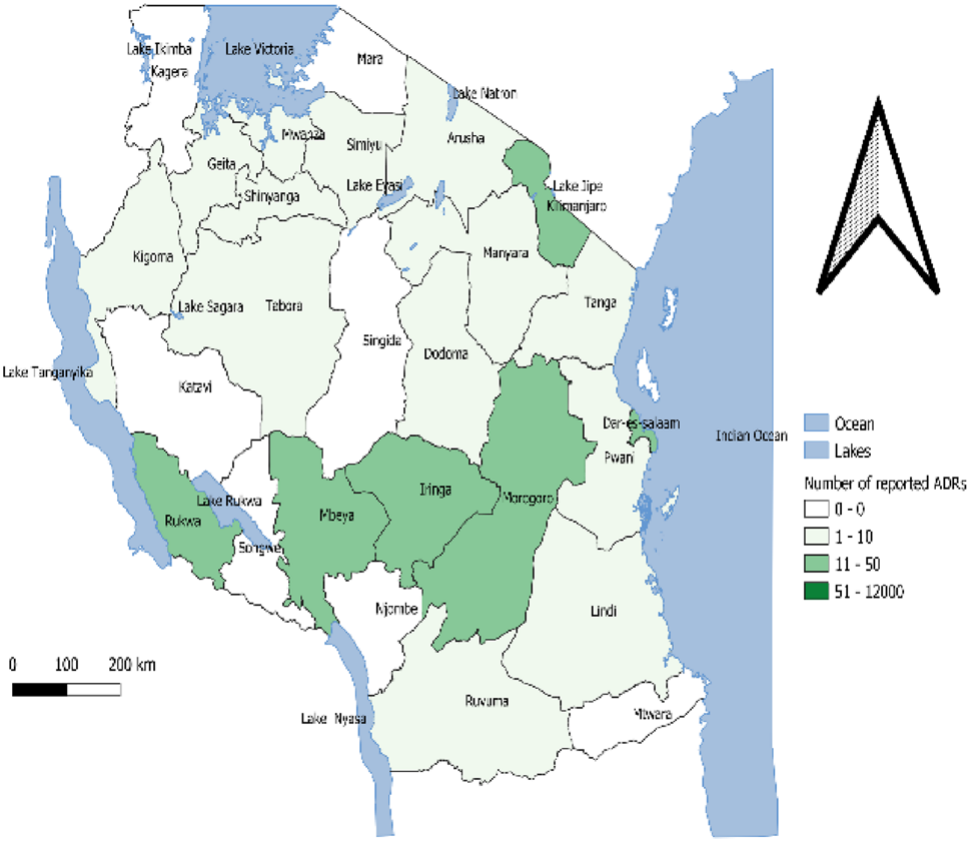
Geographical distribution of number of ADEs reports before SSSSM.

**Figure 3:**
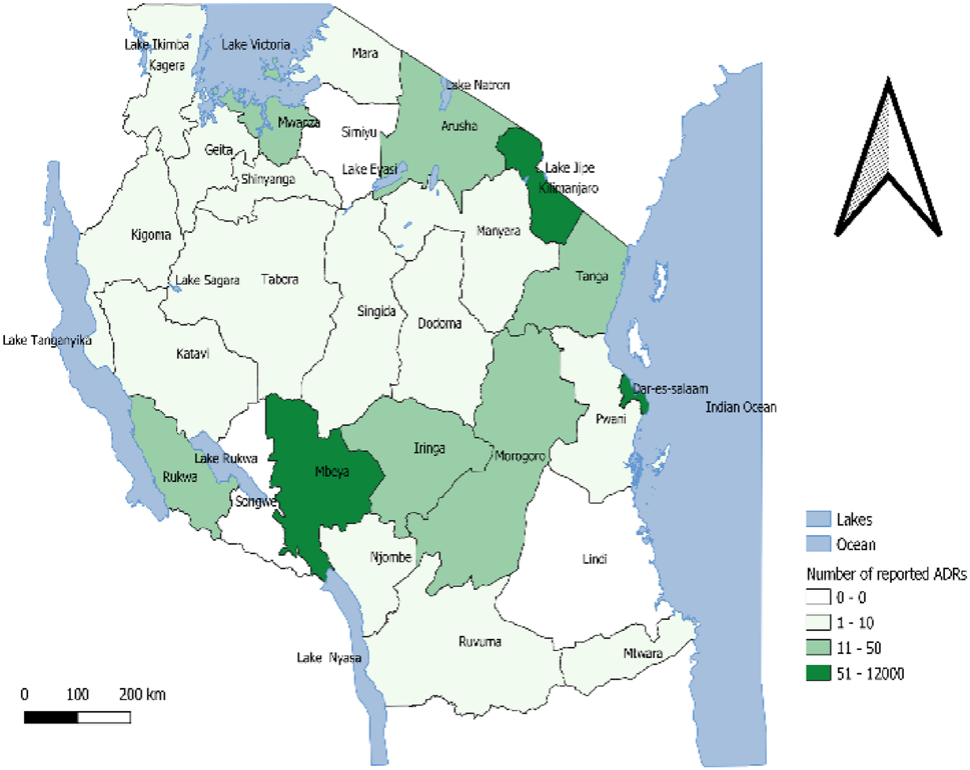
Geographical distribution of number of ADEs reports after SSSSM.

Before the implementation of SSSSM program, Kilimajaro, Rukwa, Mbeya, Iringa, Morogoro, and Dar es Salaam regions reported a high number of ADEs reports ranging from 11 to 50. However, after the implementation of the SSSSM program, there was a significant increase in the number of reports in Kilimanjaro, Mbeya, and Dar es Salaam ranging from 51 to 12,000 reports. Likewise, a slight increase in the number of reports was observed in Mwanza, Arusha, and Tanga, however, the rest of the regions did not show any changes in the ADEs reporting rate. (Fig. 2 and Fig. 3).

Maps were generated by using Quantum Geographic Information System (QGIS) version 3.10A Coruna (URL link https://gisenglish.geojamal.com/2019/11/download-qgis-310-coruna-nov-2019.html).

### Assessing the outcome of the program by identifying Signals

#### Signal detection from newly introduced medicine, Dolutegravir (DTG) based regimen

We identified a total of seven (7) ADEs that were associated with the used of Tenofovir/Lamivudine/Dolutegravir (TLD) as shown in Table 4. After signal evaluation of previous known side effect, the following were the identified previous unknown signals that were associated with the use of TLD. These signals included: weight gain (reporting odds ratio (ROR) = 16.39, 95% CI = 10.03–26.79); Increased appetite (ROR = 38.77, 95% CI = 22.19–67.69); Peripheral Neuropathy (ROR = 19.04, 95% CI = 9.36–38.75); Numbness of lower extremities (ROR = 6.68, 95% CI = 3.45–12.95); and Vaginal discharge (ROR = 2.41, 95% CI = 19.84–178.35).

**Table 4:** Signals associated with TLD after SSSSM program.

Name of ADRs of interest (At least five cases of TLD related ADRs)	Number of ADRs of interest from suspected medicine (TLD) (A)	Number of other ADRs from suspected medicine (TLD)(B)	Number of ADRs of interest from non-suspected medicine (C)	Number of other ADRs from non-suspected medicine (D)	ROR (95% CI)	*P* Value
Weight gain	21	220	95	16,316	**16.39 (10.03–26.79)**	< 0.001
Increased appetite	20	221	38	16,278	**38.77 (22.19–67.69)**	< 0.001
Diarrhoea	17	224	483	15,833	**2.49 (1.51–4.11)**	0.0002
Headache	18	223	1335	14,981	0.91 (0.56–1.47)	0.688
Itching	12	229	705	15,611	1.16 (0 .64–2.08)	0.618
Skin rash	11	230	536	15,780	1.41 (0 .76–2.59)	0.270
Peripheral Neuropathy	10	231	37	16,276	**19.04 (9.36–38.75)**	< 0.001
Numbness of lower extremities	10	231	105	16,211	**6.68 (3.45–12.95)**	< 0.001
Insomnia	9	232	259	16,057	**2.41 (1.22–4.73)**	**0.009**
Vaginal discharge	6	235	7	16,309	**59.48 (19.84–178.35)**	< 0.001

*TLD *Tenofovir/Lamivudine/Dolutegravir; *ADRs* Adverse Drug Reactions.

#### Signal detection from other medicines (Vancomycin medication error signals)

We identified a total of three (3) signals that were associated with the use of vancomycin injection as shown in Table 5. These signals included: generalized itching (reporting odds ratio (ROR) = 33.07, 95% CI = 24.43–44.76), inflammation (ROR = 33.74, 95% CI = 16.14–70.54) and swelling of face (ROR = 12.34, 95% CI = 5.53–27.55).

**Table 5:** Signals associated with vancomycin injection after SSSSM program.

Name of ADRs of interest (At least five cases of vancomycin related ADRs)	Number of ADRs of interest from suspected medicine (Vancomycin) (A)	Number of other ADRs from suspected medicine (Vancomycin) (B)	Number of ADRs of interest from non-suspected medicine (C)	Number of other ADRs from non-suspected medicine (D)	ROR (95% CI)	*P* value
Generalised itching	102	81	535	15,839	**37.27(40 -50.53)**	< 0.001
Headache	18	165	1292	15,082	1.27 (0.78–2.08)	0.332
Inflammation	8	175	30	16,344	**24 (11.25–55.09)**	< 0.001
Rash	8	175	499	15,875	1.45 (0.71 -2.97)	0.301
Swelling of face	7	176	52	16,136	1**2.34 (5.53–27.55)**	< 0.001
Fever	5	178	433	15,755	1.02 (0.42–2.49)	0.9618
Vomiting	10	173	1409	14,965	0.61 (0 .32–1.16)	0.131

## Discussion

In this study, we used a quasi-experimental design and data mining technique to assess the effectiveness of introducing the SSSSM reporting program on strengthening pharmacovigilance. The results revealed that the program increased the ADRs reporting rate. The results are in line with some studies conducted in other countries which indicated the increase in reporting rate by 59% in Denmark^24^ and 65.4% in Spain^17^ after interventions to enhance spontaneous reporting. Likewise, the systematic review conducted in 2019, education intervention increased the reporting rate from 1.02 to 70.0 folds^6^. Similar findings were reported in the studies conducted in Italy^11^, South Africa^30^, India^31^ and Brazil^32^. Authors suggested that multidisciplinary education intervention promoting changes in the participants’ behaviours and attitudes related to ADEs reporting^32^. This means that the program which includes training, sensitization, follow-up, and a structural system within the healthcare facilities contributes to an increase in ADRs reporting and hence strengthens the pharmacovigilance system.

Moreover, our results revealed that pharmacists contribute to the highest percentage of reports followed by other health professionals to embrace physicians, nurses; and consumers or non-health related professionals. Our results are different from the study conducted in Turkey which reported that physicians contributed most on reporting followed by other health professionals and pharmacists^33^. This means all healthcare providers should be trained equally for them to improve and change the attitude on ADRs reporting. In the study conducted in Nepal, it was suggested that multidisciplinary collaborative efforts focusing on increasing knowledge about PV and ADR through workshops, training, seminars, online and offline courses, and conferences might motivate HCPs in reporting ADRs^34^. Likewise, a study conducted in India suggested that to improve spontaneous reporting of ADRs it is necessary to conduct regular pharmacovigilance awareness programs, online reporting facilities, and frequent interaction with healthcare workers^31^. However, other studies have reported that, some HCPs are not reporting ADEs and among factors contributing to non-reporting among HCP includes lethargy and lack of interest^35^.

In addition, the results also indicated that MNH contributed to a large number of ICSRs compared to other hospitals. This might be due to the fact that MNH is a national hospital having many in-patients and out-patients (info@mnh.or.tz). Moreover, the hospital management committed themselves through the development of the SSSSM program to conduct all pharmacovigilance activities and set up a system involving intern pharmacists in a day and night rotation of the follow-up of patients. In addition, MNH has a higher number of intern pharmacist compared to other hospitals. At the time of data collection there were 208 intern pharmacists, while other hospitals have 2 to 20 intern pharmacists. Thus, the commitment of hospital management, setting of infrastructures for medicines safety and engagement of intern pharmacists and/or other medical professions with continuous training and sensitization may contribute to the increased number of ICSRs.

The results also indicates that, 60% of the top ten medicines reported to cause ADEs were chiral medicines. These results support the study conducted in Tanzania^23^ which showed that about 60% of all registered medicines are chiral and are more utilized as they have been listed in the National Essential Medicine List in Tanzania (NEMLT). Higher number of ICSRs with ADEs caused by methadone might be contributed by awareness and willing to report of HCWs working on methadone clinic.

Furthermore, the results indicated an increase in PBRR per 1,000,000 inhabitants from 2 reports per million in 2018 (1 year before SSSSM) to 85 reports per million in 2019 (1 year after SSSSM). These results indicate that the reports per million populations have been increased. In 2015 the analysis of spontaneous reports in the Vigibase (WHO database) revealed that the top African countries to report ICSRs per million person-years were Cape Verde (165), Namibia (119), Eritrea (104), Kenya (39), and Tunisia (32), while Tanzania had a rate of 1.68 per million person-years^36^. In the analysis of Global Patterns of Adverse Drug Reactions from 2000 to 2009, Tanzania had ADRs reporting rate of 1 reports/million inhabitants/year^37^. As compared to WHO standards, the reporting rate is still low. The WHO monitoring center in Uppsala requires each country to report a minimum of 200 reports per million inhabitants per year^17^. Our study is in line with other studies which indicated the increase of ADRs reporting rate per million inhabitants after training and sensitization on reporting. The study conducted in Turkey, ADRs reporting rate for a million inhabitants increased from 1.5 in 2005 to 32.1 in 2013 after the intervention to raise awareness and sensitizing reporting nationwide^33^. Similarly in the study conducted in Italy, ADR reporting rate increased to 230 reports per million inhabitants after local educational and editorial initiatives in 10 years^38^. In addition, it has been reported that, the increased reporting rate was due to increased awareness in post-marketing surveillance and drug safety as well as involvement of all regional pharmacovigilance centres through specific regulatory activities^38^. The analysis of ADRs conducted in Brazil indicated that, the average annual ADRs notification rate in 2008 to 2013 was 22.8 reports per million inhabitants^39^. In Portugal from 2001 to 2013, the reporting rate was estimated at 171 reports per million inhabitants^8^. In the analysis of Global Patterns of Adverse Drug Reactions from 2000 to 2009 in high-income countries had the highest ADR reporting rate ranging from 3 to 613 reports/million inhabitants/year while low-income countries had the lowest rate ranging from 0 to 21 reports/million inhabitants/year^37^. The SSSSM program conducted in Tanzania contributed to the increase in ADEs reporting rate per million inhabitants. Communication made to patients through phone calls by HCWs during the implementation of the program improved patient—HCWs relationship and awareness to report, and hence contributed to an increase in ADEs reports.

Correspondingly, the mapping of geographical differences before and after the SSSSM reporting program indicated a significant increase in the number of ADRs reports in some regions like Kilimanjaro, Mbeya, and Dar es Salaam ranging from 51 to 12,000 ADRs reports after the SSSSM program. Some regions had a slight increase in ADRs reports for example Mwanza, Arusha and Tanga. Some other regions did not show any changes in the ADRs reporting rate. These findings are in line with a study conducted in Italy which reported a noticeable difference in the number of ADRs reports across regions^38^. Previous studies have reported that lethargy, lack of interest^35^, differences in the pattern of drug use, attitudes and knowledge of pharmacovigilance and different points of view among HCPs might be the contributing factors^38,40^. Studies conducted in Denmark and UAE reported the reasons for under-reporting to be contributed by; the complexity of the reporting process and lack of reporting skills^41^, lack of time, other priorities, uncertainty concerning the drug causing the ADE, difficulty in accessing reporting forms, lack of awareness of the requirements for reporting and lack of understanding of the purpose of spontaneous reporting^24^. Likewise, other factors include unknown reporting procedures, unavailability of reporting forms, and lack of time as also reported in a study conducted in Vietnam^42^, lack of time to complete a report, lack of confidence to discuss ADRs, reporting generate extra work, concern report may be wrong and single ADR report may not affect the database as it was reported in a study conducted in South Africa^30^. Moreover, more studies should be conducted to investigate why some other regions had zero reporting despite the training and sensitization interventions after the introduction of SSSSM program.

Our study showed that the outcome of the SSSSM program was useful in detecting signals from newly introduced medicines and any medicine reported with ADEs. For the DTG-based regimen, the program identified signals that were associated with the use of TLD. These were weight gain and an increase in appetite, peripheral neuropathy, numbness of lower extremities and vaginal discharge. Previous studies also reported weight gain associated with the use of DTG-based regimens^43–45^. It was reported that the switch to a DTG-based regimen reduces insulin sensitivity which could promote storage of excess circulating glucose and lipids in adipose tissue. However, this requires further clinical study^45^. Likewise, our findings on increased appetite among patients on DTG-based regimen were congruent with analysis of data from Brazilian Pharmacovigilance center^10^. In the study conducted in Zambia, it was observed that neurological and neuropsychiatric accounted for 30% of all reported DTG-based regimen ADRs^46^. These findings were similar to our finding of presence of peripheral neuropathy and numbness signals among patients using a DTG-based regimen. However, previous studies have reported ADRs associated with the use of DTG-based regimen being headache, altered sense of balance, malaise, nausea, fatigue^46^, insomnia, anxiety, and depression^47^, diarrhoea, and headaches^10^.

The outcome of the program aided in detecting signals (related to medication error) that arose from ADRs reported after the use of vancomycin hydrochloride injection. All ADRs were reported from one facility within a short period of time (two weeks). The reported ADRs included generalized itching, inflammation and swelling of the face. Our findings were similar to previous studies which reported the main adverse effects associated with vancomycin to be hypotension, thrombocytopenia, phlebitis, nephrotoxicity, ototoxicity, hypersensitivity reactions, red man syndrome, neutropenia, chills, fever, interstitial nephritis^48–51^. In our findings the inflammation and swelling of the face were observed to be manifestations associated with red man syndrome that was caused by medication errors. Previous studies also reported the incidence of red man syndrome ranges from 3.7% to 47% in patients^50^. The authors reported a correlation of the redman syndrome with faster rates of vancomycin administration which leads to angioedema and hypotensions^50^. The program was to mitigate this medication error by training health care workers on how to administer vancomycin injection. This included slowing the infusion rate of vancomycin to 10 mg/min and premedication of antihistamines^51^.

This study demonstrates the opportunity of using data mining algorithms for signal detection in new medicines and old medicines using spontaneously reported data. This study contributes to the limited but important literature on the safety of DTG-based regimen especially in Sub-Saharan Africa and also the detection of medication errors.

## Limitations and strengths of the study

Our study has strengths and limitations of which one of the strengths is that there was no missing data. All ADEs reports were entered into the vigiflow, verified and validated and were considered during the analysis. Besides, the SSSSM program focused on seven (7) pharmacovigilance centres, other health facilities including regional and district health facilities were also trained and sensitized to the benefit of the study. Limitations are that the findings from this study should be interpreted with caution since the SSSSM program intervention was carried out in seven pharmacovigilance centres (referral and regional hospitals) which are unlikely to be representative of other health facilities. Also the before and after study design are not considered as the gold standard in evaluating the effectiveness of the intervention. However, this type of study design was considered ideal due to logistical and ethical reasons and that it was not possible to conduct a randomized clinical trial. Nevertheless, the effectiveness of SSSSM intervention on the absolute number of ADEs reports did not take into account other factors that may increase or decrease the number of ADEs reports. Moreover, the study did not take into account the quality of the reports because the aim was to increase the reporting rate after the intervention. However, we consider that the quality was good due to the training which was conducted in the pharmacovigilance centres.

## Conclusion

Our study revealed that the Structured Stimulated Spontaneous Safety Monitoring program (SSSSM) as an improved spontaneous method, can increase the adverse drug events reporting rate. This will help in early signal detection and regulatory actions to be taken to prevent further reactions to occur and hence protecting public health. The outcome of the program was also useful in detecting signals from the newly introduced medicines and other old medicines. It improved patient-healthcare relationship and increased awareness to report. This means spontaneous reporting of ADEs is still a cornerstone of pharmacovigilance, however its improvement is very essential.

## Recommendations

We recommend the SSSSM program to be implemented in all healthcare facilities. This should include continuous educational interventions and sensitizations for all healthcare providers in all facilities. The engagement of healthcare facility leaders and departments in safety monitoring of medicines will also increase the reporting. Additional educational interventions, sensitization and structural arrangements within health facilities that target ADEs reporting among HCPs are necessary for the improvement of reporting rate and may allow early detection of ADEs, prevent avoidable harms and contribute to patient safety. For sustainability in educational interventions, we recommend more focused courses in pharmacovigilance at the colleges and universities for a future generation of healthcare providers to be aware of drug safety.

## Data Availability

Dataset generated and analyzed during the study are available at TMDA office and at Vigiflow/VigiLyze (WHO website: www.vigiflow.who-umc.org / www.vigilyze.who-umc.org). All data are available with permission of Tanzania Medicines and Medical Devices Authority (TMDA).
